# Perceptions of Community Pharmacists toward the National E-Prescribing Service (Wasfaty) and Exploring the Benefits and Challenges of the Service: A Descriptive Study from Qassim Region, Saudi Arabia

**DOI:** 10.3390/pharmacy11050152

**Published:** 2023-09-21

**Authors:** Saud Alsahali, Ghazwaa Almutairi, Raghad Aedh, Sarah Alanezi, Hanan Almutairi, Mohammed Anaam, Mohammed Alshammari, Abdulmalik Alhabib, Abdullah Alowayed, Suhaj Abdulsalim

**Affiliations:** 1 Department of Pharmacy Practice, Unaizah College of Pharmacy, Qassim University, P.O. Box 5888, Unaizah 51911, Qassim, Saudi Arabia; ghazwaaalmu@gmail.com (G.A.); aiedraghad@gmail.com (R.A.); sarrah.alanezi@gmail.com (S.A.); phhananalmutairi@gmail.com (H.A.); m.anaam@qu.edu.sa (M.A.); m.alshammari@qu.edu.sa (M.A.); abdulmalikalhabib@gmail.com (A.A.); s.chalil@qu.edu.sa (S.A.); 2 Department of Pharmaceutical Care, Alrass General Hospital, Qassim Health Cluster, P.O. Box 58883, Alrass 51921, Qassim, Saudi Arabia; phabdullah09@gmail.com

**Keywords:** electronic prescribing, digital health, community pharmacists, *Wasfaty*, electronic prescription

## Abstract

Background: Electronic prescribing systems (e-prescription) for medications have many benefits, including patient safety, increase in patient satisfaction, efficiency of pharmacy work, and quality of patient care. However, few studies have been conducted to evaluate the national e-prescription system “*Wasfaty*” service in Saudi Arabia, which was recently adopted. Objective: The aims of this study were to explore the benefits observed through the use of the system and most frequent challenges experienced by community pharmacists in the Qassim region of Saudi Arabia. Methods: This study was conducted using a descriptive survey on a web-based platform. The target population of the study included community pharmacists in the Qassim region of Saudi Arabia who worked in pharmacy chains utilizing the e-prescription service between September 2022 and November 2022. Descriptive statistics along with multiple ordinal regression were used for data analysis. Results: The study population consisted of 124 pharmacists, of which 62.9% (78/124) were males and 37.1% (46/124) were females. Most of the participants had a positive perception of the e-prescription system with regard to medication safety, with 68.6% (85/124) indicating that e-prescriptions reduce the risk of dispensing errors. However, 81.5% (101/124) did not agree that the e-prescription system resulted in a reduction in workload, and 70.2% (87/124) disagreed that the service increased patient satisfaction. Conclusions: The results of this study indicated that the national e-prescription system has many benefits to healthcare employees and improves their work, particularly for patient safety, reducing medication errors, and improving the management of patient medications. The participants believe that there is a need to improve communication with prescribers, showing concern about the unavailability of some medications; thus, it is important for policymakers to encourage other pharmacy chains and suppliers to join the service to increase patient access to medications.

## 1. Introduction

Electronic prescribing systems (E-prescription) are considered to be a way to increase patient safety, the efficiency of pharmacy work, and the quality of patient care. They represent an electronic version that replaces handwritten and printed prescriptions that are generated using a computer and then sent directly to the community pharmacy [1]. The Center for Medicare and Medicaid Services (CMS) in the United States defines electronic prescribing (e-prescribing) as the transmission of prescription or prescription-related information through electronic media between a prescriber, dispenser, pharmacy benefit manager, or health plan, either directly or through an intermediary, such as an e-prescribing network. Two-way communication between the point-of-care and dispenser is an example of an e-prescribing process [2]. These systems have been established to eliminate transcribing errors caused by poorly legible documents, missing or incorrect information, and the use of non-standard abbreviations [1,3]. E-prescription systems are considered a significant tool for enhancing drug management quality, safety, and efficiency [4].

Many governments around the world have established e-prescription networks, which include regional and nationwide networks. Recently, the government of Saudi Arabia implemented a digital transformation plan for the government and private health sectors as part of Saudi Arabia’s Vision 2030 [5]. The Saudi Ministry of Health (MOH) has adopted various electronic services to facilitate administrative processes, which enable patients to book appointments and engage in online medical consultations [5]. In 2018, pharmaceutical care services at governmental hospitals and primary care centers (PHCs) underwent a major digital transformation, which enabled patients to dispense and refill medications free of charge through selected community pharmacy chains using the e-prescription system known as *Wasfaty*, which translates to “My Prescription” in English [6,7]. This allows community pharmacists who work in pharmacies that have adopted this service to play a significant role in this transformation. The pharmacists have a responsibility to provide pharmaceutical care services, including checking patient information, dispensing medications, patient counseling, and solving issues associated with patient prescriptions [6].

*Wasfaty* is an electronic system for e-prescribing services that includes prescribing by a physician in the clinic, followed by checking and dispensing medications by pharmacists at the pharmacy. The system facilitates the medication-dispensing process, ensures the availability of medications to all patients, limits unauthorized repetition and duplicate prescriptions, and saves time and resources for MOH hospitals [6]. The service begins when a prescription is created electronically by a physician in the hospital or PHC and the patient receives a notification via a short message service (SMS), including the patient ID and prescription code. The pharmacist at the community pharmacy can then access the platform to dispense the medications [6,8].

E-prescription systems are used in many countries and show many benefits for patient care, which include easy access, flexibility, saving time for the patients and pharmacists, increased availability of medications, better control over medication dispensing, and improved patient safety [1,2,8,9,10,11,12,13]. However, many challenges and limitations are evident, such as incomplete prescription information entered by physicians, technical problems, unavailable medications, and increased workload [8,9]. Because an e-prescription system represents a new service in public hospitals in Saudi Arabia to dispense medications for a large number of people involving multiple parties, there are many expected challenges that community pharmacists still face, despite the benefits of this transformation. Therefore, we used a descriptive survey to identify the benefits and to explore the most important challenges faced by community pharmacists who worked on *Wasfaty* in the Qassim region of Saudi Arabia. Moreover, we asked the participants about their general satisfaction with the new service.

## 2. Methods

### 2.1. Study Design and Setting

This study was conducted using a descriptive survey on a web-based platform. The target population included community pharmacists in the Qassim region of Saudi Arabia who worked in pharmacy chains that adopted the “*Wasfaty*” service. Community pharmacists who did not work on the “*Wasfaty*” service were not included in the survey. The population of the Qassim region at the time of the survey was approximately 1,016,000 [14]. Based on the MOH statistics, there were approximately 450 community pharmacies and 800 pharmacists that work in the private sector in the Qassim region [15]. Thus, we estimated that there were approximately 1.7 pharmacists per pharmacy, and according to the official *Wasfaty* service website [16], there were 103 pharmacies involved in services during the data collection period, and so we estimated that approximately 175 pharmacists worked in these pharmacies [15]. The sample size was calculated using Raosoft^®^ (Sample Size Calculator; Raosoft, Inc., Seattle, WA, USA), considering 5% as the margin of error and 95% as the confidence level, with a 50% response rate; thus, the minimum sample size should not be less than 121 pharmacists [17].

### 2.2. Development and Administration of the Questionnaire

A self-administered questionnaire was used, which was adapted from previous studies [3,8,9,10,13] and consisted of three sections. The first section included multiple choice questions that were used to show the participants’ demographics, number of employees in the pharmacy, years of experience, and number of daily prescriptions processed in the pharmacy. Then, we asked a question about their satisfaction with the *Wasfaty* service on a scale of 1 to 5, with 5 meaning very satisfied and 1 meaning very dissatisfied. The second part of the questionnaire was about the positive contributions of the *Wasfaty* service. By reviewing the literature [3,8,9,10,13], we detected many types of positive features for the e-prescribing system, we included 20 positive statements, and the respondents were instructed to answer using a five-point Likert scale to assess the degree of agreement: 1 = strongly disagree, 2 = disagree, 3 = neutral, 4 = agree, and 5 = strongly agree. The last section involved challenges that were experienced by the participants while using the *Wasfaty* service to show the prevalence of these challenges among them. Many types of challenges were expected to happen in e-prescription systems, including physician-related, patient-related, medication availability, and system-related challenges. For the challenge questions, the respondents were instructed to answer “Yes” or “No”. The first draft of the questionnaire was given to experts in pharmacy practice for checking its content and face validity. Then, to further improve the clarity, applicability, and understandability of the developed questionnaire, 5 community pharmacists who have experience in the Wasfaty service were invited to give feedback. Some of their feedback related to duplication in meaning in terms of some statements and minor linguistic comments in nature, so the modifications were applied based on their feedback. The responses of pilot testing were not included in the final data analysis. Then, the questionnaire was finalized and prepared for web-based using a Google form and distribution using WhatsApp (Meta Platforms, Inc., Menlo Park, CA, USA). Before data collection, the ethical approval was obtained from the Health Research Ethics Committee at Qassim University, Saudi Arabia (reference number 22-03-10). The community pharmacists working on the *Wasfaty* service in the region were invited to participate in this study through administrators at the pharmacies and with the help of their colleagues. Prior to responding to the questionnaire, the pharmacists were briefed about the aims of the survey, data protection of the participants, and were informed that participation was voluntary, and they could start filling out the survey after they had agreed. To enhance the response rate, the data collectors (R. Aedh, S. Alenazi, H. Almutairi, and G. Almutairi) approached the pharmacists working at these pharmacies to encourage them to fill in the questionnaire.

### 2.3. Analysis of the Data

The statistical package for the social sciences (SPSS) version 20.0 (IBM Corp., Armonk, NY, USA) was used to analyze the data and summarize the responses of the participants. Descriptive statistics, which included frequencies and percentages, were used to summarize the responses of the community pharmacists to the survey questions. Inferential statistics (i.e., multiple ordinal regression) were used to determine the significance of an association between pharmacists’ satisfaction versus number of years of experience, number of prescriptions processed daily, and number of employees.

## 3. Results

### 3.1. Demographics of Participants

A total of 124 community pharmacists working in the Qassim region who used the *Wasfaty* service at the pharmacy participated in this study. The population included 62.9% (78/124) male and 37.1% (46/124) female pharmacists, all of whom had bachelor’s degrees in pharmacy. The mean age was 31.29 ± 6.35 years, mean number of years of experience was 6.02 ± 5.7 years, and the number of daily prescriptions filled was 148 ± 97. The most frequent number of employees in the pharmacy was three, based on 30.7% of the participants, and the mean number of employees for all the participants was 2.97 ± 1.22. Details of the participants’ demographics are given in Table 1.

### 3.2. Satisfaction with Wasfaty Service

**Figure 1:**
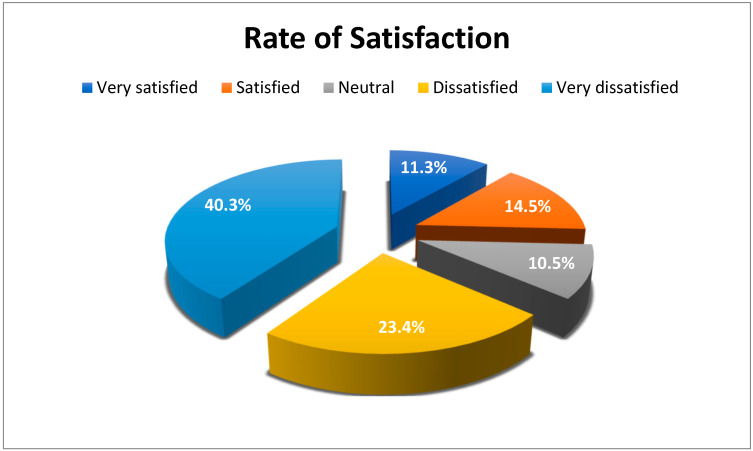
Community pharmacists’ satisfaction with *Wasfaty* service (*n* = 124).

The rate of satisfaction of the pharmacists with the *Wasfaty* service was 63.7% (79/124) dissatisfied/very dissatisfied, 25.8% (32/124) were satisfied/very satisfied, and 10.5% (13/124) were neutral, as shown in (Figure 1). The results of ordinal regression revealed an increase in pharmacist satisfaction associated with a decrease in number of years of experience (OR: 0.78; 95% CI: 0.63–0.99). There was no statistically significant association between pharmacist satisfaction with the number of employees (OR: 0.88; 95% CI: 0.62–1.25) or with the number of prescriptions processed daily (OR: 1; 95% CI: 0.99–1).

### 3.3. Positive Contributions Seen by the Community Pharmacists toward Wasfaty Service

Most community pharmacists 59.6% (74/124) acknowledged that the e-prescription system service improves the management of the drug inventory. In addition, 75.8% (94/124) of the participants agreed that the e-prescription service is associated with less ambiguities compared with traditional paper prescriptions, and 70.2% (87/124) indicated that the service helps in lowering the number of forgeries. Moreover, 70.2% (87/124) of the participants stated that e-prescriptions reduced the consumption of materials like paper and toner, and 57.2% (71/124) agreed that they promote better management of patient medications. In terms of medication safety, 68.5% (85/124) agreed that the e-prescriptions reduce the risk of dispensing errors, 69.3% (86/124) indicated that they reduce the risk of incorrect interpretations, and 61.3% (76/124) agreed to improve the monitoring of drug interactions. Nonetheless, many negative impressions of the e-prescription service were observed among the participants. For example, the highest disagreement was a reduction in workload by 81.5% (101/124), followed by a 70.1% (87/124) disagreement in increased patient satisfaction. Moreover, 63.7% (79/124) of the participants disagreed/strongly disagreed that the e-prescription service accelerate the service to the patients, and 69.3% (86/124) disagreed that the system saved time. Finally, 60% (74/124) of the participants disagreed that the service improved communication with prescribers. The details are given in Table 2.

### 3.4. Challenges Encountered by Community Pharmacists

The most prevalent challenge was the availability of prescribed medications [88.7% (110/124)]; 83.9% (104/124) indicated that the doses of medications entered were incorrect. In addition, 70.2% (87/124) of the participants reported that they encountered incorrect information entered into the system, 69.4% (86/124) reported that the physician did not fill out all the required information, 68.5% (85/124) reported there were inaccuracies in report recording, 46% (57/124) faced technical issues with the system, 55.6% (69/124) indicated that they received the wrong drug selection, and 49.2% (61/124) reported that the prescription was not recorded in the system. The details of challenges faced by community pharmacists for using *Wasfaty* are given Table 3.

## 4. Discussion

This descriptive study is one of the first studies from Qassim, Saudi Arabia, that assessed the benefits and challenges of an e-prescription system (*Wasfaty*). Most of the participants provided positive statements about the survey, particularly with respect to medication safety, reduced risk of dispensing errors 68.6% (85/124), decreased risk of the incorrect interpretation of prescription 69.5% (86/124), and the improved monitoring of drug interactions 61.3% (76/124). The results were consistent with those of previous studies that have been conducted in different areas [18,19,20,21]. In 2022, A study conducted in the Jazan Region of Saudi Arabia indicated that the *Wasfaty* service reduced prescription errors and enhanced the safety of the dispensing process [6]. Moreover, 70% (87/124) of the participants indicated that e-prescriptions resulted in fewer forgeries, which is consistent with other published studies [13,18]. As patient safety and prescription error reduction are major concerns for patient care, studies have shown that e-prescribing provides a solution to these problems; however, the e-prescription system is not free from ambiguities and errors in the dispensing process, which requires clarification from the prescribers [13].

The e-prescription system promotes better management of patient medication based on the opinions of 57.2% (71/124) of the participants. A similar percentage agreed that the system facilitates medicine tracking, and 59.6% (84/124) indicated that the system enabled them to control medication quantities for patients and the pharmacy. Many studies have shown that the e-prescription system allows pharmacists to check patient medications and to track dosages and the quantities of medication that have already been dispensed [1,6,9,18]. The improved management of patient medication may lead to better patient outcomes [18].

Regarding the reduction in workload, 81% of the participants (101/124) disagreed that the e-prescription service reduced workload. Although there are many studies showing that e-prescription systems enhance work-flow [18,22], others show that it increases the workload of the pharmacy for many reasons [23,24,25,26]. One of the main reasons is that the technology requires special training for system users, which can be costly. Also, the e-prescription system may not yet be well designed; thus, there are challenges related to technical problems and workflow issues, and connection issues remain, leading to delays in dispensing services [1,22]. These issues could lead to workflow being hindered in the pharmacy, and then patient services may affected [26]. There is also a need to hire more staff and establish training programs in pharmacies using *Wasfaty* to ensure the success of the newly adopted service based on a study conducted by Khardali et al. [6]. Moreover, the health policy regulators at the MOH need to encourage other community pharmacy chains to join the *Wasfaty* service to increase the number of service sites and to overcome transportation challenges for elderly patients [6,7].

With respect to communication between pharmacists and physicians using *Wasfay* service, 60% (74/124) of the participants disagreed that the service improved communication with prescribers. The findings of our study, related to lack of communication, were consistent with that of previous studies assessing the *Wasfaty* service among pharmacists [6] and patients [7]. In the present study, most of the pharmacists indicated that they faced challenges with recorded prescriptions, such as incorrect patient information 70.2% (87/124), physicians not filling out the required fields 69.4% (86/124), physicians not understanding the system well 69.4% (86/124), and inaccuracies in report recording 68.5% (85/124). These issues may increase the need for improved communication with physicians. A study from the United Kingdom found that an increase in communication load between physicians and pharmacists increases the workload for pharmacists. Also, the study showed that the physician relied on the technical expertise of the pharmacist for medication-related issues [27]. Another study found that some physician mistakes in medication dosage forms using the e-prescription system likely occurred because the prescriber was not required to review the final version of the prescription before sending the order to the pharmacy [28]. This indicates the need to improve the e-prescription system in terms of staff training and to design and implement efficient electronic communication between pharmacists and physicians. Also, providing technical support through the service network was important to ensure consistent workflow during dispensing processes in the service sites. This should improve the acceptance of the e-prescription system among physicians and pharmacists [28].

The participants were asked to consider whether the e-prescription system service increased patient satisfaction, and 70.1% (87/124) disagreed. The rate of satisfaction of the participants with the e-prescription application included 63.7% (79/124) that were dissatisfied/very dissatisfied. This is in contrast to studies indicating satisfaction for the e-prescription system among pharmacists and physicians [29,30]. A system with a low efficiency may increase negative perception among the users [1]. Recently, two Saudi studies evaluated pharmacists’ and patients’ experience and satisfaction with the newly adopted *Wasfaty* service. The first study by Almaghaslah et al. [7] was conducted among patients, who showed moderate satisfaction levels (score of 3.3/5) with the *Wasfaty* service. The patients indicated good satisfaction with respect to pharmacist availability, instructions given by pharmacists, and with privacy, whereas they were less satisfied with medication availability and communication between pharmacists and physicians [7]. The second study by Khardali et al. [6] interviewed pharmacists working with *Wasfaty* in the Jazan region of Saudi Arabia. Most of the pharmacists had a negative attitude towards the *Wasfaty* service for their routine work [6]. The pharmacists indicated that the service was still incomplete, inefficient, and lacked good connection between the prescribers and pharmacists [6]. Also, most of the pharmacists reported that the unavailability of medications was a major disadvantage of the *Wasfaty* service [6]. This is consistent with our findings, since most of the pharmacists in our study (88.7%; 110/124) indicated that they experienced this issue. To help address this concern, more integral work is needed between the pharmacy chains and the administration of the *Wasfaty* service to expand the list of alternative medications, as suggested in a previous study [6], and that governmental authorities should exert more efforts to ensure a consistent supply of medication in local markets in coordination with drug manufacturers and suppliers.

## 5. Limitations

This study was one of a few initial studies about the *Wasfaty* service, which is a newly launched e-prescription system designed to serve the people of Saudi Arabia. The service is developed and expanded over time, and the plan is to cover more clients in the future. Our findings showed agreement with that of previous studies; however, this study has several limitations. First, this was a cross-sectional study, so the findings only reflect a specific point in time, which may not reflect the service quality afterwards. Second, the study was conducted among community pharmacists who work with the Wasfaty service in the Qassim region of Saudi Arabia; thus, the results lack generalizability to the overall community of pharmacists throughout Saudi Arabia. Third, as a self-reporting survey, there may be some social desirability bias, which means that the participants may have tended to answer the questions about practices positively, based on what they thought was expected from them. Finally, females represented only 37.1% (46/124) of the participants, and this may be due to a lower number of female staff working at community pharmacy chains in Saudi Arabia, which was reported in a previous study. Due to gender sensitivity in local culture, more females should be hired in community pharmacies [8].

## 6. Conclusions

This study contains initial findings as well as positive results that may assist health policymakers to improve the national e-prescription system in Saudi Arabia. The community pharmacists in this study indicated that the national e-prescription system has many benefits for healthcare workers and improves their work, particularly for patient safety, reduction in medication errors, decrease prescription drug abuse, and the management of patient medications, which may result in having a positive impact on patient health and the community. Also, this study, along with others conducted around the country, has identified many areas for improvement. The national e-prescription system *Wasfaty* needs to be improved with respect to facilitating communication between pharmacists and prescribers, which may help in the quality of patient care and improve system acceptance between stakeholders. Also, there is a need for more professional training for the staff working with the e-prescription system and continued technical support at service sites. The participants were concerned about the unavailability of some medications. This problem requires more integration between pharmacy supply chains and the administration of the service to expand the alternative medications list and to notify suppliers to ensure the availability of medications in local markets. It is important for policymakers at the MOH to encourage other pharmacy chains to join the new service to increase the number of service sites for patients and to persuade pharmacy chains to hire more staff in branches using the service.

## Figures and Tables

**Table 1:** Participant’s demographic data.

Characteristics	N (124)	%	Mean ± SD (Min–Max)
Age			
20–30	73	58.9	31.29 ± 6.35 (23–52)
31–40	29	23.4	
41–50	20	16.1	
51–60	2	1.6	
Gender			
Male	78	62.9	
Female	46	37.1	
Education			
Bachelor’s degree in pharmacy	124	100	
Number of Prescriptions processed daily			
1–50	15	12.1	148 ± 97 (20–600)
51–100	24	19.3	
101–150	16	12.9	
151–200	21	17	
>200	48	38.7	
The number of people employed in the pharmacy			
1	13	10.5	2.97 ± 1.22 (1–7)
2	33	26.6	
3	38	30.7	
4	31	25	
5	5	4	
6	2	1.6	
7	2	1.6	

**Table 2:** Positive contributions seen by the community pharmacists toward *Wasfaty* Service.

Statement	Strongly Disagree	Disagree	Neutral	Agree	Strongly Agree
1. Acceleration of the service to the patient	41 (33.1%)	38 (30.6%)	5 (4%)	27 (21.8%)	13 (10.5%)
2. Saving time for pharmacist	53 (42.7%)	33 (26.6%)	7 (5.6%)	18 (14.5%)	13 (10.5%)
3. Help in eliminating the mistakes of dispensing the wrong drug	33 (26.6%)	23 (18.5%)	13 (10.5%)	30 (24.2%)	25 (20.2%)
4. Help in eliminating the difficulties which result from not being able to read the paper prescriptions	13 (10.5%)	1 (0.8%)	6 (4.8%)	44 (35.5%)	60 (48.4%)
5. Cost saving for consumable materials in dispensing process	15 (12.1%)	12 (9.7%)	10 (8.10%)	44 (35.5%)	43 (34.7%)
6. Help in workload reduction	75 (60.5%)	26 (21%)	7 (5.6%)	9 (7.3%)	7 (5.6%)
7. Help in management of inventory	23 (18.5%)	18 (14.5%)	9 (7.3%)	51 (41.1%)	23 (18.5%)
8. Help to give patients alternative medications if their medications are not available	23 (18.5%)	20 (16.1%)	5 (4%)	53 (42.7%)	23 (18.5%)
9. Help to give more time to take care of patient	35 (28.2%)	35 (28.2%)	15(12.1%)	23 (18.5%)	16 (12.9%)
10. Help in decreasing prescription drug abuse	20 (16.1%)	10 (8.1%)	17 (13.7%)	44 (35.5%)	33 (26.6%)
11. Improved communication with physicians	47 (37.9%)	27 (21.8%)	11 (8.9%)	26 (21.0%)	13 (10.5%)
12. Help to increased patient satisfaction	52 (41.9%)	35 (28.2%)	11 (8.9%)	15 (12.1%)	11 (8.9%)
13. Help facilitating medicines tracking	26 (21.0%)	10 (8.1%)	17 (13.7%)	49 (39.5%)	22 (17.7%)
14. Providing better management of the dispensed medications for the patient, which increases the quality of care for the patients	28 (22.6%)	12 (9.7%)	13 (10.5%)	49 (39.5%)	22 (17.7%)
15. Help in reducing the number of prescription forgeries	21 (16.9%)	9 (7.3%)	7 (5.6%)	56 (45.2%)	31 (25.0%)
16. Help in reducing the risk of dispensing errors	20 (16.1%)	12 (9.7%)	7 (5.6%)	59 (47.6%)	26 (21.0%)
17. Help in facilitating the monitoring of drug interactions	23 (18.5%)	13 (10.5%)	12 (9.7%)	55 (44.4%)	21 (16.9%)
18. Help in making prescriptions less ambiguous than paper prescriptions	14 (11.3%)	11 (8.9%)	5 (4.0%)	56 (45.2%)	38 (30.6%)
19. Help in the monitoring of adverse drug reactions	20 (16.1%)	12 (9.7%)	19 (15.3%)	50 (40.3%)	23 (18.5%)
20. Help in lowering the risk of the incorrect interpretation of a prescription	14 (11.3%)	13 (10.5%)	11 (8.9%)	55 (44.4%)	31 (25.0%)

**Table 3:** Challenges with *Wasfaty* service.

The Challenges Encountered by Participants	No n (%)	Yes n (%)
1. Dosage errors entered by prescriber (dose, strength)	20 (16.1%)	104 (83.9%)
2. Wrong dosage forms	56 (45.2%)	68 (54.8%)
3. Wrong medication selection	55 (44.4%)	69 (55.6%)
4. Inaccuracies in patient report recording	39 (31.5%)	85 (68.5%)
5. Inaccurate patient information entered by prescriber	37 (29.8%)	87 (70.2%)
6. The prescriber does not know a lot about the service system	38 (30.6%)	86 (69.4%)
7. The prescriber does not fill out all the required information	38 (30.6%)	86 (69.4%)
8. The patient came to the pharmacy and his/her prescription was not entered in the system	63 (50.8%)	61 (49.2%)
9. Prescribing medications that are not available in the pharmacy	14 (11.3%)	110 (88.7%)
10. Connecting challenges with the service due technical issues	67 (54.0%)	57 (46.0%)

## Data Availability

The datasets used for this study were available from the corresponding author on reasonable request.

## Abbreviations

MOH	Ministry of Health
SMS	Short Messaging Service
